# Ambiguous genitalia: An overview of 7 years experience at the Children’s Hospital & Institute of Child Health, Lahore, Pakistan

**DOI:** 10.12669/pjms.35.1.289

**Published:** 2019

**Authors:** Jaida Manzoor, Sommayya Aftab, Muhammad Yaqoob

**Affiliations:** 1 Dr. Jaida Manzoor, MBBS, FCPS. Associate Professor (Pediatric Endocrinology), Department of Pediatric Endocrinology and Diabetes, The Children’s Hospital & Institute of Child Health, Lahore, Pakistan; 2 Dr. Sommayya Aftab, MBBS, FCPS. Senior Registrar (Pediatric Endocrinology), Department of Pediatric Endocrinology and Diabetes, The Children’s Hospital & Institute of Child Health, Lahore, Pakistan; 3 Dr. Muhammad Yaqoob, MBBS, MCPS, PhD. Assistant Professor (Clinical Genetics), Department of Genetics, The Children’s Hospital & Institute of Child Health, Lahore, Pakistan

**Keywords:** Ambiguous genitalia, Congenital Adrenal Hyperplasia, Disorder of Sex Development

## Abstract

**Objective:**

To determine the classification and etiological diagnosis of children presented with ambiguous genitalia/atypical genitalia according to the newer classification system of Disorder of Sex Development (DSD).

**Methods:**

This observational, cross-sectional study was conducted at the Department of Pediatric Endocrinology and Diabetes at The Children’s Hospital &Institute of Child Health, Lahore from January, 2007 to December; 2014. Files of all the children with ambiguous genitalia were retrospectively analyzed and relevant data was retrieved. All the information was recorded on predesigned proforma and analyzed accordingly.

**Results:**

A total of 300 cases of ambiguous genitalia classified according to the new DSD classification. 46, XX DSD were 54.3% (n=163), 46, XY DSD were 43.7% (n=131), sex chromosome DSD were 2% (n=6). Among 46, XX DSD cases, the most common cause was congenital adrenal hyperplasia (97%, n=158). However, in 46, XY DSD partial androgen insensitivity/*5α-*reductase deficiency (62%. n=81) constituted the most commonest disorder. Other causes of 46XY DSD include testosterone synthesis defect(23%), congenital adrenal hyperplasia (CAH,12%), testis regression syndrome (1.5%) and persistent mullerian duct syndrome (PMDS,1.5%). Sex chromosome disorder constituted one case of iso-chromosome X turner syndrome, mixed gonadal dysgenesis (n=3), ovotesticular DSD/chimerism (n=2).

**Conclusion:**

Ambiguous genitalia have varied etiologies, 46; XXDSD found being the commonest of all, showing predominance of CAH especially salt loosing type. The early detection and prompt treatment of cases of ambiguous genitalia plays a pivotal role in the management of acute life threatening condition and gender assignment.

## INTRODUCTION

Individuals with congenital conditions in which development of chromosomal, gonadal or anatomical sex is atypical are classified as having “Disorder of Sex Development (DSD)”. Most of these patients present with atypical genitalia termed as “Ambiguous genitalia”.^1^ Previously the term Intersex was used and it was further subdivided into three categories i.e. male pseudo-hermaphrodite, female pseudo-hermaphrodite and true hermaphrodite.^2^ After consensus in 2006, new nomenclature was introduced in which the term Intersex was replaced by Disorder of Sex Development and its main categories were classified as 46, XX DSD; 46, XY DSD; and Sex chromosome DSD.^1^,^2^

Among various clinical presentations of DSD, the ambiguous appearance of genitalia is the most common disorder. The incidence of ambiguous genitalia varies between developed and developing countries and is substantially related with frequency of consanguineous marriages in a population.^3^ Studies conducted in populations with high rate of consanguinity revealed the increased incidence of ambiguous genitalia. It is supported by the fact that the incidence of ambiguous genitalia in Saudi Arabia is reported as 1 in 2,500 live births and in Egypt 1 in 3,000 live births.^3^ However, the incidence of ambiguous genitalia in Germany is low, i.e. 2 per 10,000 live births.^4^

The underlying cause of ambiguous genitalia in a newborn/child needs extensive and urgent investigations not to miss the life threatening problem like congenital adrenal hyperplasia (CAH) which comprises major presentation of DSD.^5^,^6^ Thorough clinical, hormonal, radiological, chromosomal, and molecular evaluations is therefore essentially required. However, a prompt evaluation of these assignments may be challenging for accurate diagnosis and appropriate therapy. Satisfactory management of these children does not merely demands early diagnosis and appropriate treatment but gender assignment will definitely produce a positive impact on the outcome of these children and their families.^7^,^8^

Congenital adrenal hyperplasia comprises a heterogeneous group of inherited enzymatic defects in the biosynthetic pathway of cortisol and/or aldosterone resulting in glucocorticoid deficiency, mineralocorticoid deficiency, and androgen excess.^9^ More than 75% children with CAH present with salt loosing variety at birth.^5^ These newborn have high mortality if prompt diagnosis and urgent treatment is not instituted.^5^,^6^

The purpose of this study was to classify and determine the underlying etiologies of children presented with ambiguous genitalia at a busy Pediatric Endocrine unit of tertiary care hospital endeavoring services to heavy referrals.

## METHODS

After the formal approval of study by the Institutional Review Board (IRB), all files of children with ambiguous genitalia age 0 – 16 years presented at the Pediatric Endocrine unit from 1^st^ January, 2007 to 31^st^ December, 2014 were thoroughly reviewed and essential information retrieved.

### Case ascertainment

History included age of presentation, consanguinity, family history of ambiguous genitalia, infant’s death, maternal history of drug intake during first trimester, and maternal history of virilization. Record of blood pressure, anthropometric measurements, dysmorphic features, pigmentation and complete genital examination were retrieved from the files. Pertinent investigations including karyotyping, abdomino-pelvis ultrasonography (USG) for internal reproductive organs, basal or stimulated hormonal levels (17-0H progesterone level, testosterone, dehydroepiandrosterone sulphate-DHEAS) and plasma renin assays were also noted. All information’s obtained were recorded on predesigned proforma. Children with apparently normal looking genitalia, age >16 years, precocious puberty with normal male external genitalia and incomplete file information were excluded from the study.

The information’s was analyzed to determine the different classification of DSD and varied etiologies of ambiguous genitalia were categorized as having 46,XX DSD, 46, XY DSD and Sex chromosomal DSD based upon the results of karyotyping.

### Statistical analysis

All the retrieved data was analyzed by using Statistical Package for Social Sciences (SPSS) Version 20 in table forms using as frequencies and percentages.

## RESULTS

Three hundred cases of ambiguous genitalia were enrolled in the study. Among 300 cases, 46, XX DSD were 54.3% (n=163), 46, XY DSD were 43.7% (n=131), and sex chromosomal DSD, 2% (n=6). (Table-I)

**Table-I:** Classification of cases with ambiguous genitalia in the present study according to Lee PA et al. 2006.

Diagnosis	Frequency	Percentages
46,XXDSD	163	54.3%
46,XYDSD	131	43.7%
Sex Chromosome DSD	06	2%

Total	300	100%

Among 46, XX DSD, the most common etiology was congenital adrenal hyperplasia constitutes 97% (n=158). 104 cases were found to be salt-wasting (66%, 104/158). Less common causes of 46, XX DSD include XX ovotesticular DSD (n=2), 46, XX testicular DSD (n=1), Luteoma of pregnancy (n=1) and structural defect like labial adhesions (n=1). (Table-II)

**Table-II:** Etiological classification of cases with ambiguous genitalia in the study population (n=300).

Karyotype	Etiology	Frequency (%)
46,XX DSD	Congenital adrenal hyperplasia	158 (97%)
	Salt looser	104
	Non- salt looser	54
	Luteoma of pregnancy	01 (0.6%)
	46 XX Testicular DSD	01 (0.6%)
	XX Ovotesticular DSD	02 (1.2%)
	Structural defect (Labial adhesion)	01 (0.6%)
46,XY DSD	Congenital adrenal hyperplasia	16 (12%)
	Salt looser	13
	Non- salt looser	03
	Partial androgen insenstivity /5α-reductase enzyme deficiency	81 (62%)
	Testosterone synthesis defect	30 (23%)
	Testicular regression syndrome	02 (1.5%)
	Persistent Mullerian duct syndrome (PMDS)	02 (1.5%)
Sex Chromosome DSD	45 XO/ 46XY	03 (50%)
	46XX / 46 XY	02 (33.4%)
	46,X del Y (q12)	01 (16.6%)

In 46, XY DSD with atypical genitalia, the most common cause was found to be partial androgen insensitivity syndrome (PAIS) /*5α-*reductase deficiency (62%, n=81). The second most common cause was found to be testosterone synthesis defect which constituted 23%(n=30) followed by CAH, 12%(n=16) and among these cases 80% were salt wasting (n=13). These16 cases of CAH presented with lesser virilization. Testis regression syndrome (n=2) and persistent mullerian duct syndrome (n=2) were the least common cause of 46,XY DSD. There were 6 cases of sex chromosome DSD with atypical genitalia (Table-II). Fifty one percent (n=152) cases of ambiguous genitalia presented in infancy which accounts more than half cases of our cohort had an early detection less than one year age. (Table-III)

Among 300 patients of ambiguous genitalia, 71% (n=212) showed consanguineous marriage. Consanguinity was most commonly found among CAH and PAIS /*5α-*reductase deficiency, 52% (n=156) and 10% (n=30) cases, respectively (Table-III). Family history of ambiguous genitalia was found in 56 cases among 300 patients (19%).

**Table-III:** Age at presentation and consanguinity in study population (n=300).

Diagnosis		Age of Presentation (Years)				Consanguinity	

		0 – 1	>1 – 5	>5 -10	>10-16	Yes	No
46,XX DSD	Congenital adrenal hyperplasia	100	45	07	06	148	10
	XX Ovotesticular DSD	--	--	01	01	02	--
	46XX Testicular DSD	01	--	--	--	01	--
	Luteoma of pregnancy	01	--	--	--	--	01
	Structural defect	01	--	--	--	--	01
46,XY DSD	Congenital adrenal hyperplasia	13	03	--	--	08	08
	Partial androgen insenstivity /5α-reductase enzyme deficiency	25	38	06	12	30	51
	Testosterone synthesis defect	06	19	03	02	22	08
	Testicular regression syndrome	--	01	--	01	--	02
	Persistent Mullerian duct syndrome (PMDS)	02	--	--	--	--	02
Sex Chromosome DSD	45 XO/ 46XY	02	--	--	01	--	03
	46XX / 46 XY	01	--	01	--	--	02
	46 X del Y (q,12)	--	--	01	--	01	--
	Total	152	106	19	23	212	88

## DISCUSSION

Ambiguous genitalia is a complex inherited disorder with variable presentation and different etiologies.^10^ Infants who are born with ambiguous genitalia poses a neonatal medical emergency for physical, social and psychological reasons because majority of the cases are found to be salt loosing congenital adrenal hyperplasia (75%) and undetermined sex.^6^,^10^ Therefore, thorough clinical evaluation, pertinent biochemical testing, imaging, chromosomal and molecular analysis helps in reaching correct diagnosis. Prompt evaluation requires an experienced multidisciplinary team (endocrinologists, surgeons, geneticists, neonatologists and counselors) which plays an essential role in the early management of devastating effects of adrenal crisis and gender assignment.^11^

In the present study, the majority of the cases of ambiguous genitalia presented during infancy (51%, n=152) and CAH was the commonest cause of sexual ambiguity with predominance of salt wasting variety (67%) followed by the simple virilizing defects (33%).^12^,^13^ Such results are consistent with another retrospective study conducted in Syria which revealed high prevalence of salt wasting CAH variety in 68.5%.^14^

In the present study CAH was the most common cause of ambiguous genitalia (58%, 174/300), followed by androgen insensitivity/5α-reductase deficiency (27%, n=81) and testosterone synthesis defect the least one (10%, n=30).^14^,^15^

Review of the literature uniformly shows that CAH is the most common cause of genital ambiguity among females showing 46,XX karyotype.^15^-^17^ Similar results were also observed in the present study revealing 97% of the cases with CAH in 46,XX DSD among over-virilized females. Studies have also shown that substantial proportion of children with 46,XX DSD females belong to salt wasting variety CAH, sharing similar results of present study (67%).^10^,^17^,^18^

In the present study, all cases of 46, XY DSD were under-virilized males constituting 43.7% of cases of sexual ambiguity. Sixty two percent of the patients with 46,XY DSD were suffering from PAIS/*5α-*reductase deficiency (not classified). The higher proportion of partial androgen insensitivity syndrome (PAIS) /*5α-*reductase deficiency in the preset study may be due to high rate of consanguineous marriages in this study population, and it showed comparative results of previous studies.^10^,^19^-^21^

On clinical grounds it is difficult to differentiate PAIS and *5α-*reductase deficiency but due to non-availability of specific biochemical assays (DHT: dihydrotestosterone) and molecular studies, both of these entities remain un-classified. Mutations in *AR gene* are associated with complete or partial androgen insensitivity.^22^ Complete androgen insensitivity (CAIS) usually presents without ambiguity in contrast with partial insensitivity (PAIS).^11^,^22^

Another major cause of ambiguous genitalia found in 46,XY children is testosterone synthesis defect (23%) which produces decreased testosterone level on short or prolong HCG stimulation test.^21^ We are unable to differentiate different causes of decrease testosterone production due to lack of availability of specific hormonal assays and molecular genetic analysis in our country.

In the present study 12% (n=16) of children with 46,XY DSD had CAH with under-virilization with majority were salt wasting type (n=13). This figure is also unusual high in comparative studies.^6^,^12^ Testicular regression syndrome was found in 2 children where no testes palpable with hypoplastic scrotum and short phallus. It was confirmed by raised gonadotropins level, absence of testosterone production on short and prolong HCG stimulation test and absence of testicular tissue on diagnostic laproscopy. Special molecular analysis cannot be carried out to ascertain the cause. As the index cases presented with ambiguous genitalia, it signify loss of testicular tissue occurs between 8 to 10 weeks of gestation.^23^

Consanguinity is a social factor having biological importance. In the present study 71% of children were born to mothers who were consanguineously married. When we compare this figure with the prevalence of consanguineous marriages in the general population of this region (46%), the attributable risk is 25 per 100.^24^ It shows that in future a significant number of children (25%) can be saved from CAH if such marriages are ceased. The role of consanguineous marriages on the emergence of DSD is thoroughly reviewed by Bashamboo and McElreavey.^3^

There is a meager data available on spectrum of DSD/Ambiguous genitalia in Pakistan where there is a major concern of consanguinity, and it poses a greater risk of such disorders. The present study is one of the few studies conducted in our country to explore the underlying causes of genital ambiguity in children.

In the developing countries like Pakistan where there is no newborn screening program for CAH, an early diagnosis and prompt treatment of ambiguous genitalia will prevent life threatening complications of CAH especially in highly consanguineous population. Prenatal testing and antenatal therapy for the prevention of sexual ambiguity are the future hopes in Pakistan which can be offered to the high risk families.

Moreover, there is dire need of more trained doctors, availability of specific biochemical assays, genetic testing which all help in detection of etiology and management of atypical genitalia.

## CONCLUSION

Ambiguous genitalia have varied etiologies, 46XX DSD being the commonest of all with predominance of CAH, salt wasting type. Prompt management of CAH patients not only saves the child but it also alleviates the agony of sex rearing in the family.

### Authors Contribution

**JM:** Conceived idea, designed & manuscript writing.

**SA:** Data collection, statistical analysis.

**MY:** Revised the work critically, suggestions.
